# Prognostic value of myocardial strain analysis with cardiac magnetic resonance in patients with dilated cardiomyopathy

**DOI:** 10.1186/1532-429X-16-S1-O90

**Published:** 2014-01-16

**Authors:** Sebastian Buss, Florian Andre, Kristin Breuninger, Stephanie Lehrke, Andreas Voss, Christian Galuschky, Dirk Lossnitzer, Philipp Ehlermann, Jennifer Franke, Tobias Taeger, Lutz Frankenstein, Henning Steen, Benjamin Meder, Evangelos Giannitsis, Hugo A Katus, Grigorios Korosoglou

**Affiliations:** 1 Department of Cardiology, University of Heidelberg, Heidelberg, Germany; 2 Department of Diagnostic and Interventional Radiology, DIAKO Flensburg, Flensburg, Germany; 3 Department of Psychology, University of Heidelberg, Heidelberg, Germany; 4 TomTec Imaging Systems GmbH, Unterschleissheim, Germany

## Background

Myocardial deformation analysis is an important task in the evaluation of heart failure. We and other previously showed that myocardial strain is a more sensitive marker for the prediction of cardiac events compared with left-ventricular ejection-fraction (LV-EF). The current gold standard technique to quantify myocardial deformation is CMR tagging. However, additional pulse sequences and specialized software are necessary with tagging, so that alternative ways for the estimation of strain in CMR using conventional steady-state-free-precession (SSFP) cine images would be preferable. In this regards, feature tracking is a novel tool, which can be run on conventional cine images and can help estimating myocardial strain and strain rate without the need of specialized tagging sequences. The aim was to investigate the prognostic impact of myocardial strain using feature tracking cardiac magnetic resonance in patients with dilated cardiomyopathy.

## Methods

Patients with dilated cardiomyopathy (n = 210) were examined in a 1.5T CMR-scanner. SSFP cine sequences of the three short and the three long axis views were analyzed using a prototype feature tracking software algorithm (2D CPA MR^©^, TomTec Imaging Systems GmbH). Circumferential, longitudinal and radial strains were quantitatively assessed. Patient follow-up evaluation included the composite endpoint for the occurrence of cardiac death, heart transplantation and aborted sudden cardiac death. Patients were divided in subgroups by the appearance and absence of the composite endpoint, respectively, by left-ventricular ejection-fraction (LV-EF≤35% and EF > 35%) and by the presence or absence of late gadolinium enhancement (LGE).

## Results

The predefined primary endpoint, a combined endpoint of cardiac death, heart transplantation and aborted sudden cardiac death occurred in 26 subjects during the median follow-up period of 5.3 years. Global LV longitudinal strain < -12.5% was a significant predictor of survival. Using multivariable analysis global longitudinal strain exhibited an independent prognostic value for the composite endpoint surpassing the value of NYHA functional class, NT-proBNP, LV-EF, global LV radial and circumferential strain as well as LGE (HR = 1.23, p < 0.05). Reduced global longitudinal strain (≥-12.5%) was strongly predictive for worse outcomes even in patients with non-severely impaired LV-EF (≥35%; HR = 0.025, χ^2 ^= 37.9, p < 0.001) and in those without LGE (HR = 0.12, χ^2 ^= 12.7, p < 0.001). Global longitudinal strain < -12.5% on the other hand, predicted favorable outcome even in patients with severely impaired EF < 35% (HR = 0.21, χ^2 ^= 7.9, p < 0.01) and in those with LGE (HR = 0.07, χ^2 ^= 21.2, p < 0.001).

## Conclusions

Myocardial strain calculation using CMR feature tracking in patients with dilated cardiomyopathy is related to survival and may aid the risk stratification of such patients independent of age, clinical parameters and established cardiac risk markers as the presence of LGE and reduced LV-EF.

## Funding

None.

